# Sleep Disorders in Stroke: An Update on Management

**DOI:** 10.14336/AD.2020.0707

**Published:** 2021-04-01

**Authors:** Hongxia Cai, Xiao-Ping Wang, Guo-Yuan Yang

**Affiliations:** ^1^Department of Neurology, Tong-Ren Hospital, Shanghai Jiao Tong University School of Medicine, Shanghai, China.; ^2^Med-X Research Institute and School of Biomedical Engineering, Shanghai Jiao Tong University, Shanghai, China.

**Keywords:** stroke, sleep disorders, treatment strategy, post-stroke depression

## Abstract

Stroke is a leading cause of disability and mortality all over the world. Due to an aging population, the incidence of stroke is rising significantly, which has led to devastating consequences for patients. In addition to traditional risk factors such as age, hypertension, hyperlipidemia, diabetes and atrial fibrillation, sleep disorders, as independent modifiable risk factors for stroke, have been highlighted increasingly. In this review, we provide an overview of common types of current sleep disturbances in cerebrovascular diseases, including insomnia, hypersomnia, breathing-related sleep disorders, and parasomnias. Moreover, evidence-based clinical therapeutic strategies and pitfalls of specific sleep disorders after stroke are discussed. We also review the neurobiological mechanisms of these treatments as well as their effects on stroke. Since depression after stroke is so prevalent and closely related to sleep disorders, treatments of post-stroke depression are also briefly mentioned in this review article.

Sleeping is one of the most important physiological processes of our body [1, 2]. Approximately 20-40% of one day is sleep time, and during this period sleep exerts multiple functions in our body. For example, it relieves mental fatigue, improves memory, regulates metabolism, and plays key roles in tissue regeneration, synaptic stability, and immune regulation [3, 4]. Numerous detrimental influences of sleep disorders have been described: decreased physical capacities (fatigue, dizziness, injuries, and mortality), psychiatric symptoms (anxiety, depression, and mania) and impaired cognitive abilities (low memory recall and learning abilities and dementia) [4]. Despite the damage mentioned above, we are forced to curtail sleep time due to social and work-related activities. If the synchronization between the outside surroundings and circadian rhythms is lost or if the rhythm itself fails to work, sleep disorders will happen [5]. Sleep disorders are mainly classified as insomnia, hypersomnia, sleep-related breathing disorders, circadian rhythm sleep-wake disorders, sleep-related movement disorders, parasomnias, and other sleep disorders. Each diagnosis possesses specific coding information [6, 7]. During recent years, evidence of sleep disorders in neurological diseases is growing rapidly [8, 9]. Several diseases such as Parkinson’s disease, multiple system atrophy and dementia with Lewy bodies (DLB) display various sleep disturbances including rapid eye movement (REM) sleep behavior disorder (RBD), insomnia and restless leg syndrome (RLS) [10]. Apart from these neurodegenerative diseases, one common neurological disease—stroke, has also been found to be closely associated with sleep disorders.

Stroke is a detrimental disease that affects 15 million patients worldwide each year, accounting for one-third of patient deaths and two-thirds of severe disabilities [11]. Despite the declining stroke incidence and death rates over the past 20 years, the number of survivors after stroke is still not increasing [12, 13]. Typical stroke symptoms are characterized by paralysis, diplopia, ataxia, numbness, and vertigo. For severe stroke, patients present with bilateral blindness, quadriplegia, unconsciousness, or even death [14, 15]. Although stroke as a heterogeneous disease with over 150 known causes, it is generally classified by cerebral ischemia and hemorrhage, subarachnoid hemorrhage, cerebral venous thrombosis, and spinal cord stroke [16, 17]. Among them, ischemic stroke accounts for about 80% [11, 18]. Classical stroke risk factors mainly include carotid stenosis, hypercholesterolemia, high blood pressure, and atrial fibrillation. In addition to these factors, other possible risk factors include migraine, chronic kidney disease, and diabetes mellitus [14, 19]. Various unhealthy lifestyles such as a high-fat diet, physical inactivity, cigarette smoking, environmental pollution, alcohol or drug abuse, and emotional stress also contribute to stroke events [12, 20]. Since sleep disorders play key roles as both a risk factor and a factor in stroke recovery and prognosis [21], in this review, we will mainly discuss several stroke-related sleep disorders as well as their treatment strategies.

## Insomnia and stroke

Insomnia, as a prevalent clinical problem, indicates sleep reduction and difficulty in starting or maintaining sleep. Before the diagnosis, the patients should provide a complete sleep history, recent physical and emotional state, and medication usage. More than 30 minutes and over 3 times per week of awakening complaints should be considered insomnia. Symptoms lasting less and more than 3 months are defined as short-term and chronic insomnia, respectively [10].

As high as approximately 50% of patients may present with insomnia during the first few months after stroke. One-third of patients present with insomnia for the first time, and the remaining patients already suffered from it previously [22, 23]. Insomnia after stroke is usually due to environmental factors or comorbidities such as depression, which will be mentioned in the latter part of the review. Particularly, insomnia may be directly associated with brain injury [21]. It was reported that ponto-mesencephalic stroke resulted in a near-complete loss of sleep, and thalamic stroke led to a lack of brain waves [22]. It was also reported that supratentorial, left hemispheric or paramedian thalamic stroke decreased non-REM (NREM), while right hemispheric stroke decreased REM [24]. In addition, insomnia was also related to damage to some specific areas of the cerebral cortex in patients with penetrating brain damage [25]. Moreover, several drugs used to treat stroke, or its comorbidities may affect sleep. For example, stroke patients with hypertension take medications such as beta-blockers, clonidine or diuretics, which can disrupt REM sleep, induce insomnia, and lead to early morning awakening, nightmares or painful calf cramps during sleep. For stroke patients with psychiatric symptoms, they would take selective serotonin reuptake inhibitors (SSRIs) such as sertraline or paroxetine, which decrease REM sleep and increase daytime fatigue [10]. Furthermore, insomnia might be the potential risk factor for stroke. A study found that short sleep (less than 5 to 6 hours per night) could reliably predict stroke after adjusting for confounding factors [26]. One cohort study including 21,438 insomniacs and 64,314 noninsomniacs suggested that insomniacs group had a 54% higher stroke risk than noninsomniacs, which was evident among young adults. The mechanisms by which insomnia may lead to stroke development are still unknown, although neuro-inflammation may play a role. Furthermore, chronic stress from sleep loss may be another possible mechanism of elevated stroke risk [21, 27]. For patients with acute stroke, insomnia was not only associated with poor life satisfaction but also with severe stroke since sleep absence can aggravate brain damage and impede neurogenesis [28, 29].

## Treatments of insomnia in stroke

For the treatment of insomnia, stroke patients should prevent themselves from experiencing noise and light (remaining in quiet rooms at night). One recommended treatment is cognitive-behavioral therapy (CBT). CBT can be applied in patients who need long-term management of chronic insomnia. It seems to reduce insomnia effectively after stroke. However, the long-term physical and mental benefits do not seem to be apparent and need to be addressed with larger samples in the future [30, 31].

For pharmacological therapy, benzodiazepines are not recommended since they may aggravate breathing-related sleep disorders as well as induce the reappearance of motor deficits. It was reported that antidepressants such as mianserin may improve insomnia after stroke [32, 33]. Zolpidem, which has relatively fewer effects on cognition and muscle tension, might improve stroke prognosis by increasing brain-derived neurotrophic factor (BDNF) secretion and protecting the neurovascular unit in acute stroke [21, 34, 35]. Nevertheless, we should be aware that hypnotics also increased stroke risk in some researches. Evidence shows that zolpidem is related to ischemic stroke risk with increasing doses, and annual use of over 4 grams or 95 days use of benzodiazepines increased stroke incidence [36]. Furthermore, the use of hypnotics has been related to medication dependence and elevated the risk of falls when the elderly walked to the toilet at night. Other problems such as impulse control disorders including gambling, shopping, or eating might also come up [10]. Therefore, a risk-safety balance needs to be further assessed before utilizing hypnotics to treat post-stroke insomnia.

Several randomized controlled trials have suggested that acupuncture (Shen-Men and Nei-Kuan acupoints) is a helpful tool for patients with insomnia after stroke (Table 1) [37, 38]. One study indicated that with the Pittsburgh sleep quality index (PSQI) evaluation, stroke patients with insomnia receiving acupuncture (Shen-Men and Si-Shen-Chong acupoints) tended to exhibit more effective outcomes than those taking drugs [39]. However, other trials have suggested that acupuncture stimulation (Da-Zhui and Sheng-Shu acupoints) shows similar therapeutic efficacy to specific hypnotics [40, 41]. All these findings suggest that acupuncture is a promising method for treating insomnia after stroke. Based on these outcomes, further large multicentre studies to confirm the efficacy of acupuncture in treating post-stroke insomnia are needed.

**Table 1:** Randomized clinical trials on treatments of insomnia or hypersomnia after stroke.

Study	Trial Type	Patients	Number Enrolled	Treatments	Primary Outcome
Nguyen et al [31]	Single-centre, parallel two-group pilot randomized trial	Stroke, insomnia	15 (intervention:9; control: 5)	8 weekly sessions of CBT and 6 continued rehabilitation.	Insomnia was improved instantly after therapy (ISI 3.89, 95% CI: 0.65 to 7.14) but was no longer superior to control after follow-up (ISI 5.25, 95% CI: -0.80 to 11.30)
Palomaki et al [32]	Randomized, double-blind, controlled trial	Acute ischemic stroke, insomnia	100 (intervention: 51; control: 49)	Mianserin 60 mg/d or placebo for 1 year, follow up for 6 months.	Mianserin treatment showed beneficial effects on insomnia recovery in stroke patients (OR: 4.04, 95% CI: 1.33 to 12.2).
Kim et al [37]	Randomized controlled trial	Hospitalized stroke, insomnia	Final 30 (intervention: 15, control: 15)	Acupuncture intradermally for 2 days.	Real acupuncture improved insomnia more than sham acupuncture (P<0.05).
Lee et al [38]	Randomized, double-blind, crossover trial	Hospitalized stroke, insomnia	Final 52 (intervention: 27; control: 25)	Acupuncture of Shen-Men and Nei-Kuan for 3 days.	Acupuncture was useful and decreased sympathetic hyperactivities for post-stroke insomnia (P<0.05).
Tang et al [40]	Randomized controlled trial	Stroke, insomnia	Final 119 (intervention: 40; medication control: 40; placebo control: 39)	Low-frequency electric stimulation on Da-Zhui and Sheng-Shu acupoints once per day for 30 days.	Acupoint stimulation and estazolam had a similar effect, and both were better than the placebo group (95.0% versus 92.5% versus 17.9%).
Hou et al [41]	Randomized controlled trial	Stroke, insomnia	90 (intervention: 30; medication control: 30; placebo control: 30)	RTAS for 40 min once a day; 2.5 mg diazepam per day; placebo capsule per day for 1 month.	RTAS was as effective as diazepam in treating insomnia after stroke, and both were better than the placebo group (86.7% versus 90.0% versus 20.0%).
Li Pi Shan et al [133]	Randomized, double-blind, crossover trial	Stroke or brain-injured, insomnia	Final 18 (9 received treatment A, 9 received treatment B)	Treatment A: 0.5-1.0 mg lorazepam for 7 days +3.75-7.5 mg zopiclone for 7 days; Treatment B: reverse order of A.	Zopiclone showed the same effect as lorazepam on insomnia in stroke or brain-injured patients (P>0.05).
West et al [49]	Quasi-randomized, controlled trial	Stroke, sleepiness and fatigue	Final 71 (intervention: 39; control: 32)	Rehabilitation in a unit with naturalistic light; the control with standard lighting for more than 2 weeks.	No improvement of hypersomnia in the intervention group (PSQI: -13.0%, 95% CI: -37.1% to 20.5%).

Abbreviations: CBT: cognitive behavior therapy; CI: confidence interval; ISI: Insomnia Severity Index; OR: odds ratio; PSQI: Pittsburgh sleep quality index; RTAS: repetitive transcranial acupuncture stimulation.

## Hypersomnia and stroke

For patients, frequent symptoms of hypersomnia are excessive daytime sleepiness (EDS) and fatigue. Fatigue can persist for years, while EDS improves during the first month after stroke [21, 42]. Hypersomnia mostly appears after subcortical and ponto-mesencephalic stroke. Stroke in the paramedian thalamus is one of the most classic forms of post-stroke EDS. These lesion sites affect the pontine tegmental reticular formation and the paramedian nuclei of the thalamus, facilitating hypersomnia formation [43]. Furthermore, large cerebral lesions and lesions involving the left hemisphere and the anterior regions also result in increased hypersomnia [24]. Patients exhibit severe hypersomnia behavior accompanied by lack of attention, memory and cognition, possibly due to damage to specific physiological functions of the sleep-wake cycle [44, 45]. In general, EDS improves within months, while other defects such as cognitive problems persist. For some patients with bilateral stroke, hypersomnia may even last for years [21, 42].

Hypersomnia also affects stroke. Studies have shown that sleeping over nine hours per night strongly predicts stroke events after eliminating confounding factors [26]. It has also been reported that excessive sleep is associated with a higher risk of stroke than short sleep [46]. A cross-sectional analysis (1,244 stroke-free participants recruited for MRI) found that subcortical white matter hyperintensities were correlated with long sleep, suggesting that hypersomnolence might be attributed to small vessel disease of the brain [47]. Furthermore, hypersomnia impeded stroke recovery, and patients had more disability and more chances of referral to convalescent hospitals [48]. The potential mechanisms were elusive, but abnormal lipid metabolism, inflammation and atherosclerosis might be involved [46].

## Treatments of hypersomnia in stroke

Therapy for post-stroke hypersomnia is full of challenges. One study reported that sleep structures in thalamic stroke survivors were ameliorated by taking medications such as modafinil or methylphenidate [44]. It was also reported that satisfactory effects on neurological recovery were observed after methylphenidate or levodopa treatment. These might be partly related to ameliorated wakefulness [22]. Antidepressants may also improve hypersomnia. West et al suggested no evident improvement in natural light interference compared with standard lighting in the rehabilitation unit (Table 1) [49]. High-quality randomized clinical trials are still lacking. Whether hypersomnia treatment improves stroke prognosis and recurrence risk is unclear and needs to be revealed.

## Breathing-related sleep disorders (BSDs) and stroke

According to the diagnostic and statistical manual of mental disorders of the 5^th^ edition (DSM-5), BSDs include obstructive sleep apnea (OSA), central sleep apnea (CSA), sleep-related hypoventilation, and circadian rhythm sleep-wake disorders [50]. The most frequent screening questionnaires on BSDs are the Berlin questionnaire (BQ), Epworth sleepiness scale (ESS), and STOP-BANG questionnaire (SBQ). The polysomnography (PSG) assay is regarded as the optimal diagnostic standard for OSA and accurately monitors capillary oxygen saturation, nasal airflow and respiratory movements [51]. BSDs are severe in acute cerebrovascular diseases. An investigation on the population with acute stroke and transient ischemic attack (TIA) revealed that BSDs were related to patients with wake-up stroke and structural defects of the heart. The probable explanation might be a paradoxical embolism resulting from BSDs [21]. In a systematic review of stroke and TIA patients, 10 studies all reported that BSD increased vascular events or mortality [52]. BSDs might improve with stroke recovery but elevated mortality continuously after the acute stage [53, 54].

Among BSDs, OSA is the most prevalent form. Obstructive upper airway induces paroxysmal hypoxia and leads to OSA during the process of sleeping. The palate and tongue are the primary blocked locations [22, 53]. In addition, an abnormal airway pressure change appears after airway obstruction. Hypoxia is accompanied by altered intrathoracic pressure, blood pressure fluctuations, and sympathetic activation, with possible mechanisms of endothelial dysfunction, oxidative stress, atherosclerosis, cardiac arrhythmia, hypercoagulation, paradoxical embolisms, and heart failure. All these events could induce stroke; therefore, OSA is a known risk factor for cerebrovascular diseases [55]. Studies have shown that approximately 4-7% of the adult OSA population suffers from cerebral ischemia and cardiovascular death, and approximately 60% of stroke patients suffer from OSA [22, 56, 57]. OSA also has negative impacts on the recovery and recurrence of stroke, whether from short or long-term observations [58]. One study reported that sleep apnea was closely associated with mood depression, delirium, and impairment in reaction and activities of daily living (ADL) abilities [59, 60]. In OSA, abnormal pathways such as paroxysmal hypoxia and autonomic activation are triggered and ultimately affect atrial fibrillation and cerebral vaso-regulation [61]. Nocturia, one common problem for senior stroke patients, is becoming a warning of severe OSA and reflects the tendency of cerebrovascular diseases [62]. In OSA patients, the lesion sites of stroke (both ischemic and hemorrhagic) were distributed in the cerebral hemispheres, brainstem and cerebellar areas [63]. Therefore, it seemed unrelated to specific infarct lesion sites in patients with OSA.

CSA possesses lower morbidity than OSA, but it is prevalent in specific populations with cardiac and cerebrovascular diseases. CSA is characterized by a syndrome of periodic airflow decline or interruption dysregulated by central ventilation. Weak respiration lowers blood oxygen levels and may finally induce stroke or heart failure. Thus, CSA is an independent stroke risk factor and improves with stroke recovery. Furthermore, CSA is negatively correlated with stroke prognosis [64]. The brainstem is the most frequently described stroke position in patients with CSA [65]. It was found that frontal lobe stroke is related to respiratory apraxia, pontine stroke to central hypoventilation, inferomedial posterior pons stroke to apneustic respirations, and medullary stroke to Ondine’s curse [24]. Cheyne-Stokes breathing has been reported to be the primary type of CSA but seems unrelated to specific lesion sites [59, 66]. In rare cases, stroke patients might suffer from complex sleep apnea (both OSA and CSA), which is likely to be neglected [66, 67].

**Table 2:** Randomized clinical trials on treatments of OSA after stroke.

Study	Trial Type	Patients	Number Enrolled	Treatments	Primary Outcomes
Bravata et al [73]	Randomized home-based study	OSA, stroke and hypertension	225 (intervention: 110; control: 115)	Auto-titrating CPAP for 1 year.	CPAP improved OSA but did not lower blood pressure compared with control (-1.1 mmHg, 95% CI: -4.2 to 2.0).
Minnerup et al [76]	Randomized, open-label, parallel-group trial	OSA, acute ischemic stroke	50 (intervention: 25; control: 25)	CPAP on the 1^st^ night after stroke onset for 3 nights; Another 4 nights of CPAP were added if AHI was over 10/hour.	CPAP was practicable on the 1^st^ night of stroke and did not aggravate the outcome (reduced AHI: 32.2±25.3 to 9.8±6.6).
Knot et al [74]	Pilot randomized, double-blind, sham-controlled trial	OSA, stroke in the rehabilitation	Final 30 (intervention: 13; sham control: 17)	Active CPAP use for median 14 days.	A benefit trend of CPAP was found in stroke recovery (median change in FIM: 34 versus 26, p = 0.25).
Bravata et al [75]	Randomized controlled trial	OSA, acute ischemic stroke and TIA	252 (control: 84; standard: 86; enhanced: 82)	CPAP on average 50% of nights, similar among standard and enhanced patients over 1 year.	CPAP improved neurological functions in patients with OSA and acute cerebral ischemia/TIA (mRS:33 versus 19 versus 28; NIHSS: 32 versus 19 versus 27).
Parra et al [77]	Prospective, randomized controlled and multi-centre study	OSA, ischemic stroke	Final 126 (intervention: 57; control: 69)	Nasal CPAP treated for 2 years and measured at 1, 3, 12, and 24 months.	Early CPAP use accelerated recovery but did not improve their survival or life quality (Rankin scale: OR: 7.78; 95% CI: 1.73 to 39.84).
Gupta et al [78]	Randomized, parallel group trial	OSA, stroke	Final 70 (intervention: 30; control: 40)	Post-titration nightly CPAP treatment, following up 3, 6, and 12 months.	CPAP group had better stroke outcomes but without a significant difference in the recurrence of vascular events (3.3% versus 15%, P=0.23).
Ryan et at [69]	Randomized, open-label, parallel-group trial	OSA, stroke patients in rehabilitation units	Final 44 (intervention: 22; control: 22)	At least 6 hours of CPAP per night for 4 weeks.	CPAP improved motor function but not cognitive outcomes compared with baseline (SART: P=0.21 versus P=0.32).
Sandberg et al [71]	Randomized controlled trial	Sleep apnoea, 2-4 weeks post-stroke	Final 59 (intervention: 31; control: 28)	CPAP treatment for 4 weeks.	CPAP decreased depression but not delirium or cognition in stroke patients with sleep apnoea (MMSE: 2.6; 95% CI: 1.1 to 4.1).
Aaronson et al [79]	Randomized controlled trial	OSA, stroke patients in rehabilitation units	36 (intervention: 20; control: 16)	CPAP treatment for 4 weeks, following up for 2 months.	Cognitive function was improved in the CPAP group (P<0.05).
Hsu et al [82]	Randomized controlled single-blind trial	OSA, stroke	Final 30 (intervention: 15; control: 15)	CPAP treated for 8 weeks, measuring at 8 weeks and 6 months after stroke.	CPAP showed no benefit in neurological outcomes or sleepiness, and some even in poorer health status (P>0.1).
Svatikova et al [87]	Randomized, controlled, crossover study	OSA, first 14 days of ischemic stroke	18 (intervention: 9; control: 9)	Positional therapy (therapeutic pillow on the first or second night) for 3 months.	Positional therapy reduced OSA severity after ischemia and improved outcomes (AHI was reduced by 19.5%, 95% CI: 4.9% to 31.9%).
Wheeler et al [85]	Randomized, controlled, two-period crossover study	OSA, acute ischemic stroke	Final 19 (intervention:11; control: 8)	Repeated measures of EPAP administered every other day.	EPAP was not superior to CPAP (AHI difference: -5.43; 95% CI: -16.6 to 5.76, P=0.314).
Jiang et al [91]	Randomized controlled trial	Sleep apnea, ischemic stroke	53 (intervention: 29; control: 24)	Mechanical ventilation (NTS) in the test group and CPAP in the control group.	NTS showed a better prognosis of respiratory function and neurological recovery than CPAP (reduce NIHSS and Barthel scores, P<0.05).

Abbreviations: AHI: apnea-hypopnea index; BSD: breathing-related sleep disorders; CI: confidence interval; CPAP: continuous positive airway pressure; EPAP: expiratory positive airway pressure; FIM: functional independence measure; mRS: modified Rankin Scale; NIHSS: national institutes of health stroke scale; NTS: nasal endotracheal suction; OR: odds ratio; OSA: obstructive sleep apnea; PSG: polysomnography; SART: sustained attention response time; TIA: transient ischemic attack.

## Treatment of BSDs in stroke

Continuous positive airway pressure (CPAP) is the first choice for OSA treatment [68]. There are different reports on the outcomes of CPAP treatment on OSA. Most studies have suggested beneficial effects, particularly in sleepiness, depression, functional recovery, and recurrent events [69-72]. A home-based randomized trial on managing sleep apnea in chronic stroke patients showed that CPAP use at home improved OSA symptoms compared with non-use (Table 2) [73]. Two randomized controlled studies also proved that CPAP was superior to conventional therapies in terms of neurological recovery [74, 75]. The results of the study by Minnerup et al showed that CPAP utilized in the first night after stroke was practicable and was not associated with worsened outcomes [76]. Several trials supported the positive efficacy of CPAP but also pointed out some limitations. For example, Parra et al reported that early initiation with CPAP promoted recovery and postponed vascular events, but it did not improve patients’ quality of life [77]. Gupta et al proved that the CPAP group displayed no apparent advantages in the recurrence of vascular events despite its favorable stroke prognosis [78]. Ryan indicated that CPAP treatment improved motor function but not neurological cognition, and Sandberg suggested that CPAP therapy did not aggravate cognitive decline in stroke patients [69, 71]. Contrary to these results mentioned above, Aaronson et.al believed that CPAP treatment improved cognitive function in stroke patients with OSA [79]. The distinction was possibly due to the CPAP treatment duration, which needs further standardization. Other investigations indicated no evident differences in the outcome, but some beneficial aspects of CPAP such as daytime wakefulness and low recurrent stroke rates were mentioned [80, 81]. However, reports from Hsu et al suggested that CPAP treatment could not improve stroke outcomes or sleepiness, and in some cases, even worsened health conditions [82].

Due to the increasingly positive evidence on CPAP therapy, the American Heart Association stated that CPAP should be applied to individuals with cerebral ischemia and TIA accompanied by OSA [83]. Consistent with the suggestion mentioned above, one recent analysis of CPAP intervention suggested that once CPAP treatment was tolerated, it was acceptable after stroke, strengthening its advantageous position for brain recovery [84]. However, less than half of patients could tolerate the device [10]. Expiratory positive airway pressure (EPAP) showed better compliance but was reported to be no better than CPAP in terms of treatment effects [85]. Symptoms such as severe swallowing disorders, depression, and dementia problems in patients could help to increase their CPAP compliances [86].

Though not systematically examined, weight loss should improve OSA. Weight loss could reduce the apnea-hypopnea index (AHI), which is determined by the sums of hypopnea and apnea presented during sleeping times [10]. OSA severity can be judged by the AHI and over 30 events of apnea/hypopnea in one hour should be diagnosed as severe OSA [51]. Svatikova suggested that random positional therapy (therapeutic pillow) after stroke led to a reduced AHI compared with no positional therapy [87]. Another first-line treatment is changing lifestyles such as physical activity and dieting. Keeping records of sleep information daily for 14 days was recommended for patients having difficulty falling or staying asleep [10, 51]. Moreover, preventing and treating secondary complications such as pain and respiratory infections should always be included in patient management. Drugs such as depressants and substances including ethanol should be used cautiously due to their harmful effects on sleep breathing.

As a second-line therapy, surgical treatment is reasonable for patients unable to tolerate CPAP. Surgical methods mainly include hypoglossal nerve stimulation (HNS), upper Cairway surgery, nasal reconstruction, and mandibular advancement [88, 89]. One trial revealed that in contrast with CPAP and placebo, the surgery group showed a beneficial tendency in life and sleep quality, though without significant differences [90]. A randomized trial from Jiang et al showed that nasal endotracheal suction exhibited better breathing function and brain recovery than CPAP [91]. However, one opposite finding was that no obvious improvement after surgery compared with CPAP [92]. Randomized clinical trials of treatments of OSA after stroke are illustrated in Table 2, and further investigations are required due to the limited evidence of the effect of therapy of OSA after stroke.

Until now, published randomized trials of CSA treatment were mainly performed in patients with heart disease. Bradley et al reported that CPAP treatment alleviated CSA and cardiac functions but did not improve survival in heart failure [93]. Cowie et al reported that adaptive servo-ventilation (ASV) was useless in the recovery of heart failure or was even associated with increased mortality [94]. Another trial showed that ASV used in patients with CSA and systolic cardiac failure did not affect heart function [95]. By biphasic positive airway pressure (BiPAP) treatment, Noda et al found that patients with dilated cardiomyopathy and CSA displayed favorite outcomes on left ventricular function [96]. For the treatment of CSA after stroke, despite few randomized clinical trials, we found some reports. One retrospective analysis indicated that patients with brain ischemia receiving CPAP or BiPAP treatments failed to control CSA expectantly. When they were switched to the ASV therapy, beneficial outcomes appeared [97]. Other results also suggested that CPAP might not be the best choice in stroke patients with hypocapnic CSA and that ASV could be helpful in normocapnic CSA. However, for patients with hypercapnic CSA or hypoventilation, mechanical ventilation should be taken into account [65, 97]. In heart failure, patients with both OSA and CSA were treated with CPAP in some cases, but ASV was a more feasible option in the long run [98]. Whether these results could be applied to stroke patients need further research. Furthermore, some drugs (antidepressants, benzodiazepines) taken by stroke patients might negatively influence CSA and should be avoided if possible [99, 100].

## Parasomnias and stroke

In the DSM-5, parasomnias include non-rapid eye movement sleep arousal disorders, nightmare disorder, RBDs, and RLS [50]. Among them, RBD and RLS are the most prevalent sleep disorders associated with stroke.

For patients with RBD, they play dreams into real actions during REM sleep with their muscle tension lost and phasic muscle activities elevated. Patients with RBD might suffer from neurodegenerative diseases, and these populations are prone to suffer from stroke later on, suggesting a correlation between RBD and stroke. A cohort study including 12,003 participants showed that probable RBD was associated with a higher risk of developing stroke (both cerebral ischemia and hemorrhage), suggesting that RBD was a neglected stroke risk factor. Increased vascular stress due to sympathetic overactivity might be one of neurobiological mechanisms [101]. One observational study of 119 patients with brain ischemia reported that the RBD prevalence was approximately 11%. It was reported that about 46% of RBD patients suffered from brainstem infarcts. Patients with RBD displayed a higher likelihood and proportion of acute brainstem infarct than those without RBD, suggesting that brainstem may be the primary lesion position in stroke patients with RBD [102]. RLS is characterized by an impulsion to exercise the limbs. Patients feel comfortable when doing some activities. However, at rest or at night, their symptoms are worsened. The main complaint of RLS is its abnormal sense of dragging, itching, formication, or pain. RLS has been reported to be correlated to diabetes, Parkinson's disease, iron deficiency, and terminal kidney disease, and has a tendency of autosomal-dominant transmission [10]. Studies have shown that RLS can induce insomnia and is often accompanied by periodic limb movements during sleep (PLMS), which involve periodically repeated moving limb involuntarily during NREM sleep [103, 104]. Transiently activated sympathetic nerves are increased in patients with RLS-related PLMS. In these patients, sleep quality declined. Whether the autonomic reaction can trigger high blood pressure should be investigated [22]. RLS occurs in about 12% of post-stroke patients. Among them, 30% have unilateral RLS symptoms, and 70% have bilateral symptoms [104]. In stroke patients, PLMS is a more frequent symptom than RLS. Stroke-related RLS primarily involves the basal ganglia. The body of the caudate nucleus is damaged, which increases dopaminergic tone and leads to RLS [105]. One case report showed that RLS appeared after the right lenticulostriate region infarction, suggesting a role of subcortical regions such as the basal ganglia-brainstem system in the regulation of motor behaviors and awake-sleep states [104, 106]. Based on two cohort studies with a follow-up period of over 8 years, risk factors for stroke such as high blood pressure predicted RLS events, followed by further stroke occurrence in larger studies [107, 108]. Patients with RLS had thicker necks, higher diabetes prevalence and poorer sleep quality than stroke patients without RLS [103]. Furthermore, stroke recovery was undesirable in the RLS group by evaluation of the modified Rankin scale and Barthel Index. Though RLS has been proven to be related to stroke prevalence, we could not determine a causative relationship between RLS and stroke [109]. A study of 346 stroke patients showed that RLS could predict subcortical stroke, suggesting a potential role as a stroke risk factor [110]. Since available evidence is inadequate to unveil the exact correlation between RLS and stroke, more conclusive studies are pressingly needed.

**Table 3:** Neurobiological mechanisms of current treatments and their effects on stroke.

Sleep disorder	Treatment	Biological mechanisms	Effects on stroke
Insomnia	CBT	Decreasing dysfunctional beliefs and attitudes [134]	Effective in patients with post-stroke depression [135]
	BZD	Hypnotic and sedative effects by binding to GABA_A_ to lower neural excitability, but aggravate OSA [136]	Both neuroprotection (low dosage) and neurotoxicity (high dosage) [36]
	Mianserin	Tetracyclic antidepressant and anxiolytic effects by antagonizing histamine and serotonin receptors [137, 138]	Delaying neuronal death on ischemic stroke animal model [139, 140]
	Zolpidem	Hypnotic by inhibiting GABA receptor [141, 142]	Increasing BDNF secretion and protecting the neurovascular unit in acute stroke but harmful at high doses [35]
	Acupuncture	Reducing the sympathetic nervous activity [38]	Debatable influence on stroke rehabilitation, warrant further studies [143, 144]
Hypersomnia	Modafinil	Weak dopamine re-uptake inhibitor to promote awakening [145]	Reducing fatigue and improved quality of life after stroke [146]
	Methylphenidate	A norepinephrine-dopamine reuptake inhibitor and neurological stimulant [147]	Improving ischemic stroke recovery slightly [148]
	Levodopa	A precursor of the dopamine, modulating sleep-wake state [22]	Improving functional recovery after stroke [149]
OSA	CPAP/EPAP	Improving obstructive breathing and hypoxia state [150]	Generally beneficial outcomes for stroke patients [73, 77]
	Positional therapy	Changing sleep position and ameliorate hypoxia [87]	Improving stroke outcomes [87]
	Surgery	Airway reconstruction to benefit ventilation [88]	Better brain recovery [91]
CSA	ASV/mechanical ventilation	Improving hypoventilation and maintaining blood-gas balance [65]	Beneficial outcomes in stroke patients [97]
RBD	Clonazepam	Binding GABA receptor and inhibiting neural excitability [151]	Improving the outcome of post-stroke movement disorders [152]
	Melatonin	Activating melatonin receptor to decrease violent attacks and improve dream enactment [21]	Brain protection after stroke [153, 154]
	Fluoxetine	Inhibiting serotonin re-uptake and playing antidepressant effect [155]	Enhancing motor recovery in ischemic stroke [156, 157]
RLS	Levodopa and dopamine agonists	Activating dopaminergic pathway and regulating extrapyramidal system [158]	Improving functional recovery after stroke [149]
	Gabapentin	Inhibiting voltage-dependent calcium channels, improving sensory and motor symptoms [159, 160]	Neuroprotection, seizure and neuralgia suppression after stroke [161, 162]

Abbreviations: ASV: adaptive servo-ventilation; BDNF: brain-derived neurotrophic factor; BZD: Benzodiazepines; CBT: cognitive-behavioral therapy; CPAP: continuous positive airway pressure; CSA: central sleep apnea; EPAP: expiratory positive airway pressure; GABA: gamma-aminobutyric acid; OSA: obstructive sleep apnea; RBD: rapid eye movement sleep behavior disorder; RLS: insomnia and restless leg syndrome.

## Treatments of RBD and RLS in stroke

First and foremost, both injury prevention and prognostic counseling should be included in RBD management. Currently, primary and secondary RBD are treated similarly. The results from one case report showed that symptoms were relieved by clonazepam in 2 patients with RBD after stroke [30]. Doses of clonazepam ranging from 0.25-2.0 mg were reported to be effective for treating stroke patients with RBD [21, 30]. In addition to clonazepam, melatonin can decrease violent attacks and improve dream enactment. Fluoxetine was also effective in stroke-associated RBD but was reported merely in one individual with syncope [111]. Medications including alcohol, stimulants, SSRIs, and selegiline worsened RBD and should be avoided. Since most proof is based on case reports, reliable randomized clinical studies on pharmacotherapy should be emphasized in the next step.

It is well established that levodopa, dopamine agonists (pramipexole and ropinirole) and gabapentin can be used to treat stroke-related RLS/PLMS patients [104]. In general, dopamine agonists, though associated with more side effects, played more effective roles than levodopa, since significant relief was reported in most patients taking dopamine agonists. In addition, the rate of spontaneous improvement observed was approximately 25%, indicating that some patients can go without pharmacotherapy [21, 112]. Iron supplementation is an important step of treatment in patients with iron deficiency. Several drugs including antidepressants and diazepam should be used with caution due to their adverse effects on RLS. The neurobiological mechanisms of current treatments and their effects on stroke are summarized in Table 3.

As the primary subtype of stroke, ischemic stroke is the most commonly researched cerebrovascular disease together with sleep disorders. However, there were no significant differences in the frequency of sleep disorders among patients with cerebral ischemia and hemorrhage. Moreover, sleep disturbances were slightly more frequent in hemorrhagic stroke [113]. One large population-based study including 95,023 adults showed that sleep duration over 8 hours was closely associated with cerebral hemorrhage in women. Men who slept over 10 hours had a tendency of higher mortality of hemorrhagic stroke [114]. Furthermore, OSA was reported in a patient with intracerebral hemorrhage [115]. Sleep and wake disorders were also found in patients after subarachnoid hemorrhage [116]. For RBD, deposition of α-synuclein could be related to amyloid angiopathy and intracerebral hematoma formation, suggesting that RBD increased the risk of hemorrhagic stroke [101]. Similarly, unilateral or asymmetrical RLS might pre-exist in patients with subcortical hemorrhage, suggesting its predictive value for hemorrhagic stroke [110]. Taken together, a body of evidence suggests that sleep disorders are strongly related to not only ischemic but also hemorrhagic stroke events. In other words, sleep disorders play roles as both risk factors for and complications of hemorrhagic stroke [117]. Therefore, hemorrhagic stroke patients should be paid the same attention as ischemic stroke patients, and proper management should be performed in the long run.

## Sleep disorders and post-stroke depression (PSD)

As one mental complication of stroke, depression has been the focus of research and received more attention than other complications such as anxiety. PSD usually presents during two ranges after stroke: one between 3-6 months and the other between 2-3 years after stroke [118]. With the same clinical manifestations of traditional depression, PSD is mainly characterized by mood dysfunctions including depressive feelings, indifference, irritability, and vegetative signs including sleep disorders [119, 120]. Despite the similarity to other depressed patients with atherosclerotic diseases, the symptoms of depression were more severe in patients with PSD than in those with atherosclerotic diseases [121]. It was recorded that the number of patients currently being examined for PSD was over 7,000 and that the prevalence of PSD was about 30% in stroke survivors [122, 123]. The percentage fluctuated with the research population, depression severity, and follow-up durations enrolled by researchers. Though prevalent, PSD was often underestimated, and some patients remained unrecognized and untreated, which further increased the morbidity. Studies have revealed that PSD predicts mortality and is connected with stroke occurrence and recovery [124, 125].

One cross-sectional study examined the connection between sleep and PSD. It was found that PSD was correlated with poor sleep quality and subjective parameters of sleep [126]. Another research on elderly patients in China showed that neural dysfunction and insomnia were related to PSD [127]. For patients with PSD in the acute phase of stroke, sleep disorders were common but nonspecific, and fatigue and inappetence might be key predictors of PSD [118]. Hence, for stroke patients, PSD and sleep disorders are closely interrelated with each other and must be actively treated together.

Since the treatments of sleep disorders after stroke are mentioned above, immediate therapy for PSD is very helpful for improving long-term recovery of stroke as well as sleep disturbances after stroke. One survey found that patients with PSD taking antidepressants from the first month of stroke presented more improvement in daily living activities than those taking antidepressants after one month of stroke. Furthermore, the early-used group maintained alleviated symptoms for up to 2 years. This finding indicated that early treatment of PSD was related to improvement in physical activities and stroke recovery [128]. For PSD therapy, it was recommended to use antidepressants such as SSRIs, which could improve depression with time [129, 130]. In addition to SSRIs (such as citalopram), nortriptyline and reboxetine have also been widely researched, but their contraindications and plasma concentrations should be carefully noted [122]. Considering the intricate complications of stroke individuals, optimal balances between the benefits and adverse effects of drugs are needed. Other methods such as CBT, electroconvulsive therapy, and psychological treatments are promising but need more evidence [122, 131].

**Figure 1.**
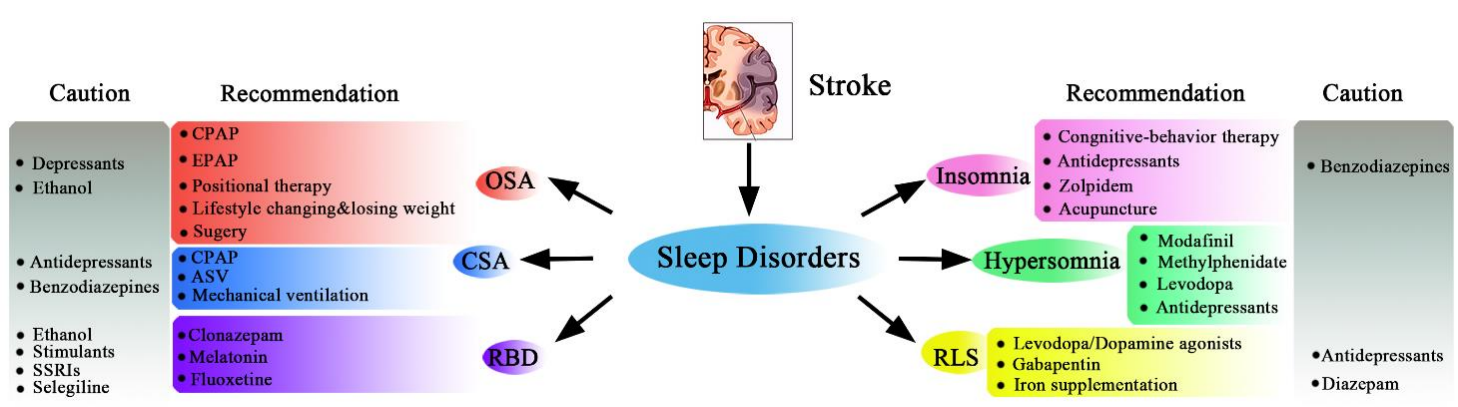
Summary of therapeutic recommendations and cautions of sleep disorders after stroke. ASV: adaptive servo-ventilation; CPAP: continuous positive airway pressure; CSA: central sleep apnea; EPAP: expiratory positive airway pressure; OSA: obstructive sleep apnea; SSRIs: selective serotonin reuptake inhibitors; RBD: rapid eye movement sleep behavior disorder; RLS: restless leg syndrome.

## Future perspectives and conclusion

To date, scientists have explored different types of sleep disorders in stroke and have put forward potential treatment strategies for them as well as pitfalls of these treatments (Fig. 1). However, unresolved questions still exist. Research on sleep disorders in animal models of stroke can help us further deeply understand how the brain functions to control sleep and wake since basic research is revealing more pathophysiological mechanisms underlying sleep disorders and intricate relationships between stroke and sleep processes. It has been proven that stroke related-sleep disturbances negatively regulate angiogenesis, axonal sprouting and synaptogenesis, aggravate brain damage and ultimately hinder neurological recovery [28, 132]. However, from clinical perspectives, there is still limited literature researching the causal relationships between specific sleep disorders and stroke recovery although sleep disorders remain as both a risk factor for and poor outcome of stroke [68]. Meanwhile, more attention should be paid to large randomized controlled trials of post-stroke sleep disturbances such as hypersomnia, RLS and RBD. Moreover, detailed investigations on the correlations of sleep disorders and PSD, their treatments and possible prognosis are pressingly needed.

In conclusion, we focused on specific therapeutic interventions of sleep disorders in stroke. Briefly, OSA treatment with CPAP is recommended in view of increasingly supportive proof. Oxygen, ASV, or non-invasive ventilation may be considered in patients with CSA after stroke. Treatments of insomnia with sedative antidepressants, hypersomnia with stimulants, RLS with dopaminergic drugs, and RBD with clonazepam are based on limited reports and should be adopted individually. Behavioral-neuropsychological therapy and other supporting therapies such as acupuncture are promising but need more convincing evidence. Furthermore, sleep disorders with PSD should also be emphasized and treated properly. In the future, we confidently believe that sleep disorders in stroke patients will be paid more attention to and that more individualized and evidence-based management will be developed.
